# Supplementary figures and images for: Analysis of the Leishmania mexicana promastigote cell cycle using imaging flow cytometry provides new insights into cell cycle flexibility and events of short duration
Source: PLoS One. 2024 Oct 3;19(10):e0311367. doi: 10.1371/journal.pone.0311367 (PMC11449296; doi:10.1371/journal.pone.0311367)

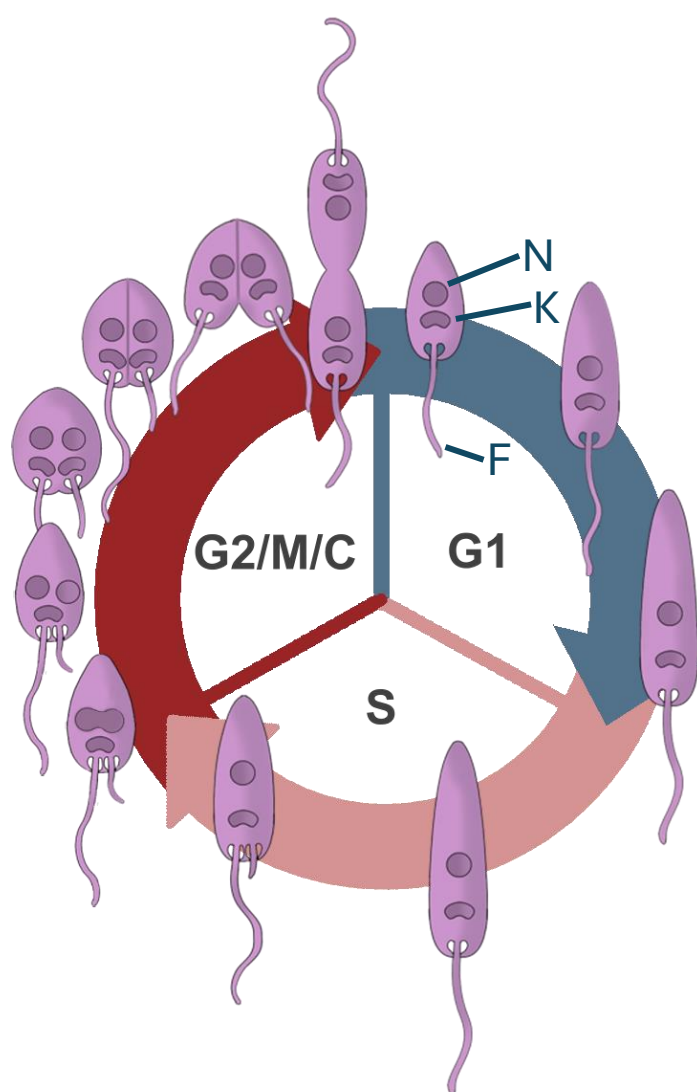

**S1 Fig**

Supplement: S1 Fig — A representation of the morphological changes seen as a cell progresses through the different cell cycle stages. The relative timings of the cell cycle stages are not to scale. M: mitosis; C: cytokinesis; N: nucleus; K: kinetoplast; F: flagellum. (PDF) [file pone.0311367.s001.pdf]

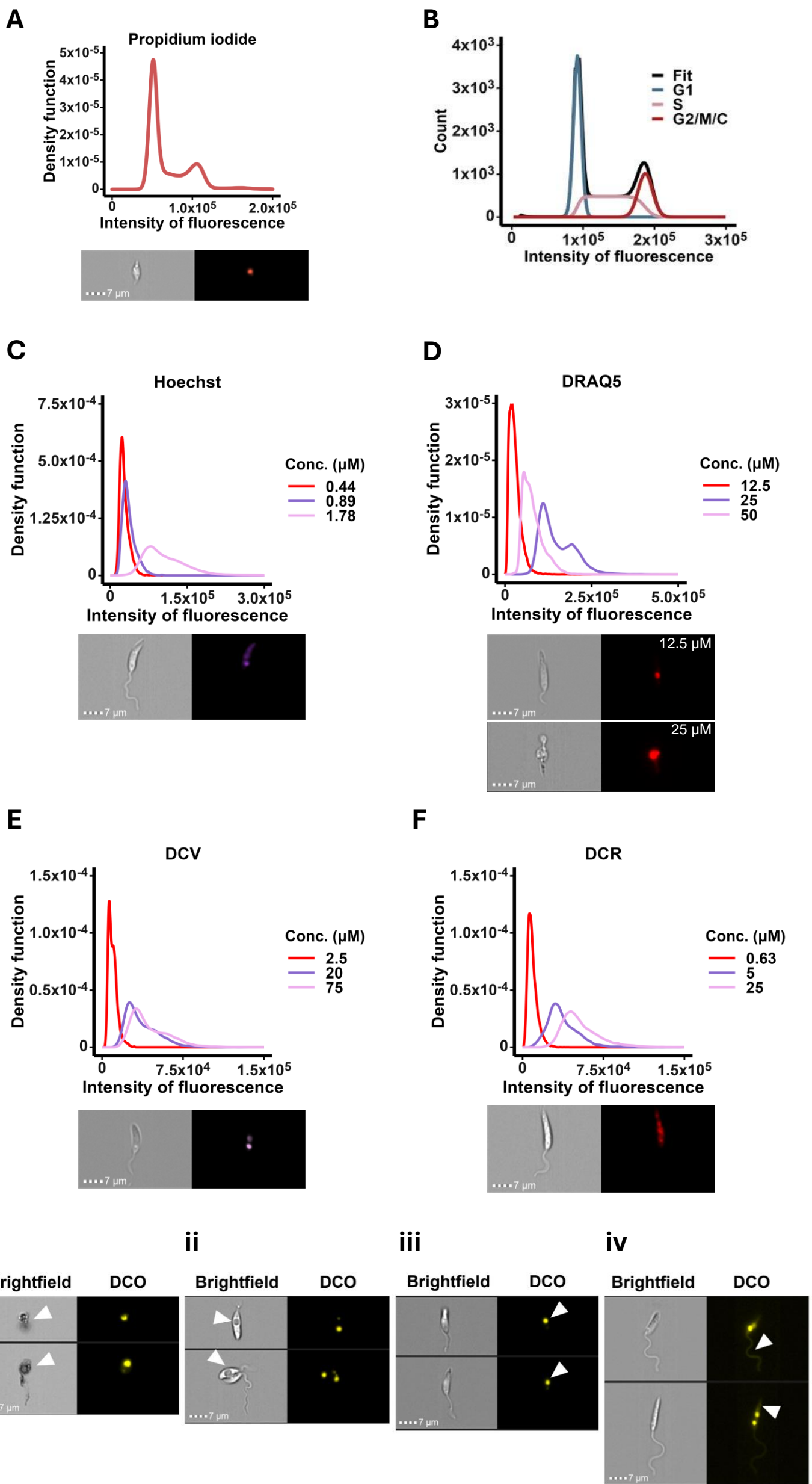

S2 Fig

Supplement: S2 Fig — C9T7 cells were incubated with various dyes, as detailed in the Materials and Methods, before being analysed on the ImageStream. Each intensity profile shows the data acquired from ~30,000 cells, with representative images below (left panel: brightfield image; right panel: fluorescence image; scale bars 7 μm). (A) Methanol-fixed cells stained with propidium iodide. (B) Cell cycle modelling of the propidium iodide DNA intensity profile (panel A) using the FCS Express™ Multicycle engine (Rabinovitch & Bagwell debris subtraction [43, 44] and Dean/Jett/Fox cell cycle modelling [45]). The cell cycle stages of each curve are indicated. C-F: live cells stained with three different concentrations of Hoechst, DRAQ5, Vybrant™ DyeCycle™ Violet (DCV) and Vybrant™ DyeCycle™ Ruby (DCR), respectively. (G) Representative images (left panels: brightfield image; right panels: fluorescence image; scale bars 7 μm) of artefacts obtained when staining with suboptimal concentrations of DCO (1.25–10 μM) at room temperature or 27°C. At a concentration of 10 μM, toxicity was observed with alterations to cell morphology (i) and the appearance of vacuoles (ii). At lower concentrations (1.25–5 μM), suboptimal staining was observed with either just the nucleus (and not the kinetoplast) staining (iii) or staining being observed additionally in the cytoplasm and/or cell membrane (iv). (PDF) [file pone.0311367.s002.pdf]

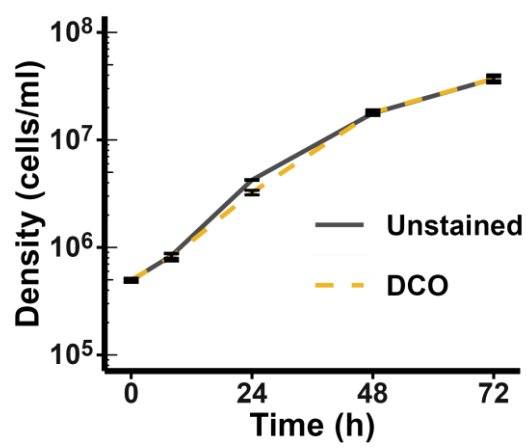

**S3 Fig**

Supplement: S3 Fig — Following incubation with DCO, C9T7 cells were washed three times in PBS and resuspended in M199 at a density of 5 x 10^5^ cellsml-1. Control (unstained) cells were similarly processed. Cell density was measured over 72 hours and a growth curve plotted. (PDF) [file pone.0311367.s003.pdf]

A

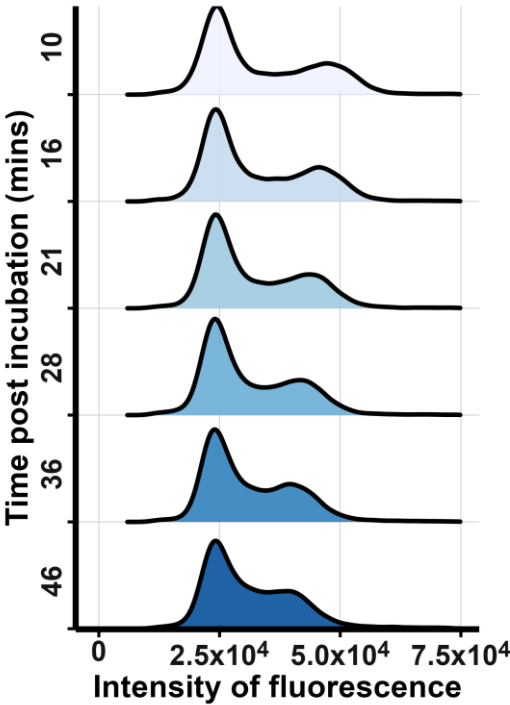

B

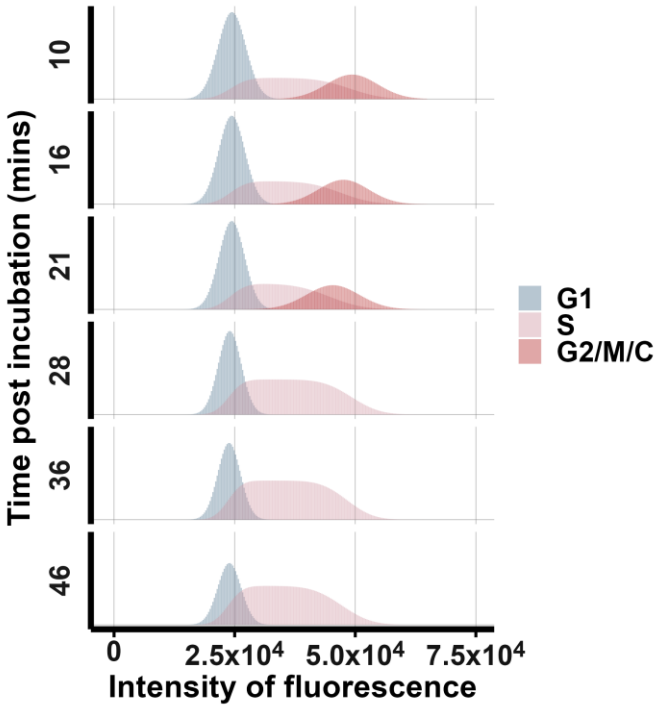

S4 Fig

Supplement: S4 Fig — (A) Triplicate populations of L. mexicana promastigote C9T7 cells were stained with 0.625 μM DCO for 30 minutes at room temperature before being resuspended in PBS. Following resuspension in PBS, cell samples were analysed by IFC at the time points indicated. Fluorescence intensity profiles are shown for one representative replicate (n = ~15,000 cells). (B) Cell cycle modelling of the fluorescence intensity profiles presented in (A) using FCS Express™. (PDF) [file pone.0311367.s004.pdf]

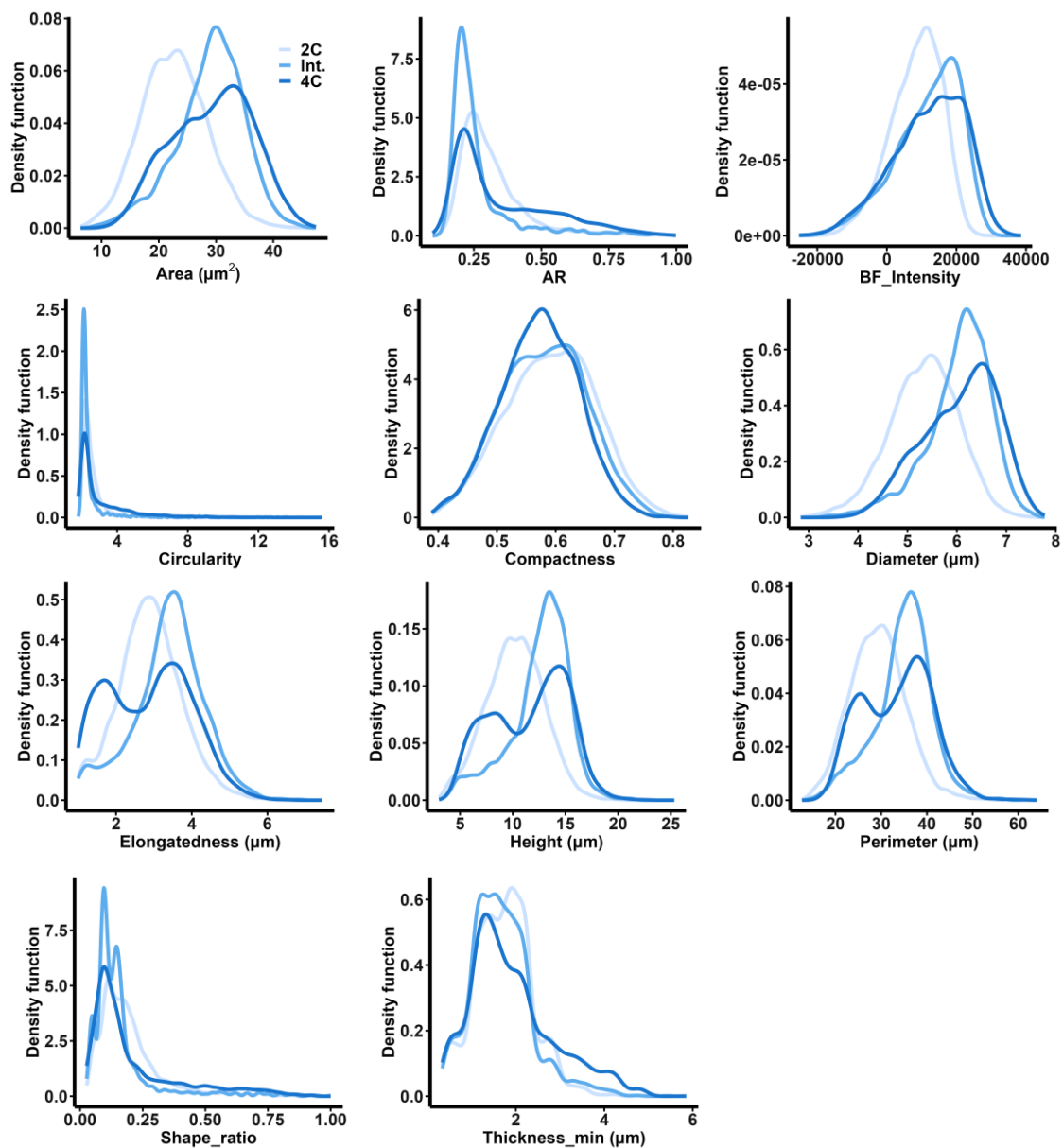

**S5 Fig**

Supplement: S5 Fig — L. mexicana C9T7 promastigotes were stained with DCO, analysed by IFC and gated according to their DNA intensity (2C, intermediate DNA content (Int.; 2C-4C) and 4C) (Fig 3A). The area, aspect ratio (AR), brightfield intensity (BF_Intensity), circularity, compactness, diameter, elongatedness, height, perimeter, shape ratio and thickness_min of the cells were then plotted for each gate, as indicated in the figure. (PDF) [file pone.0311367.s005.pdf]

**A**

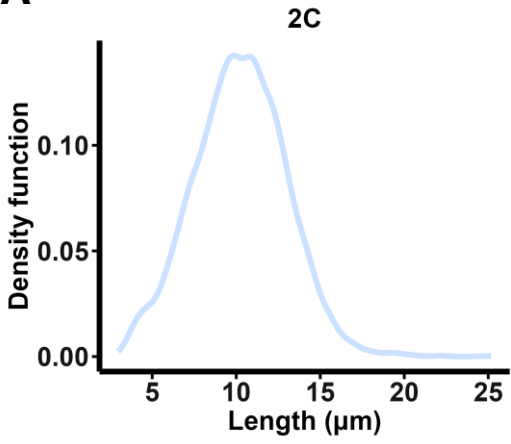

**B**

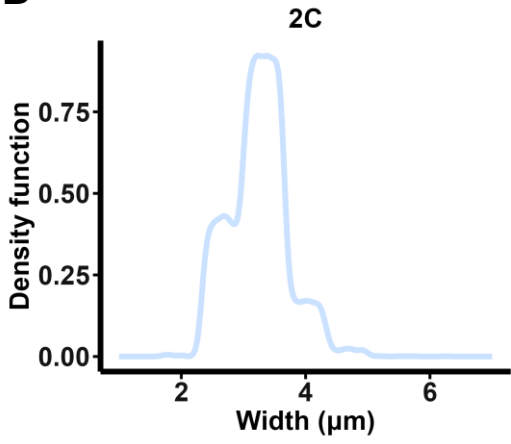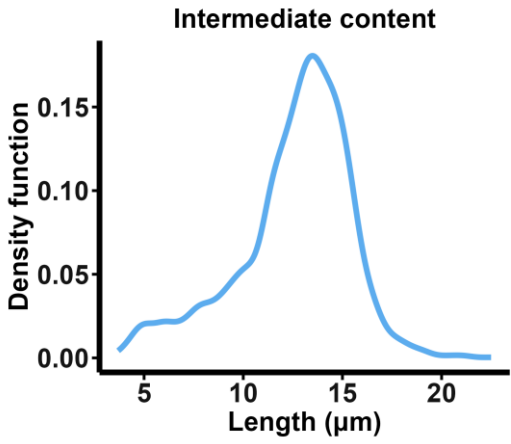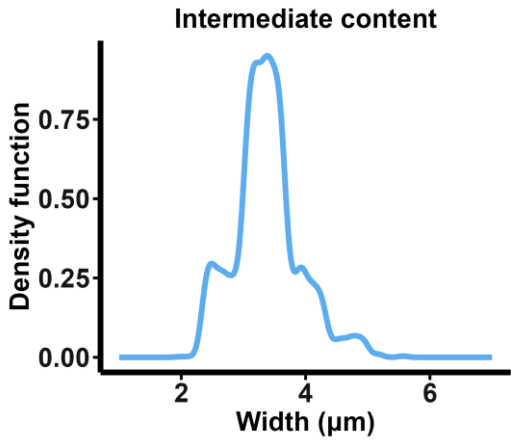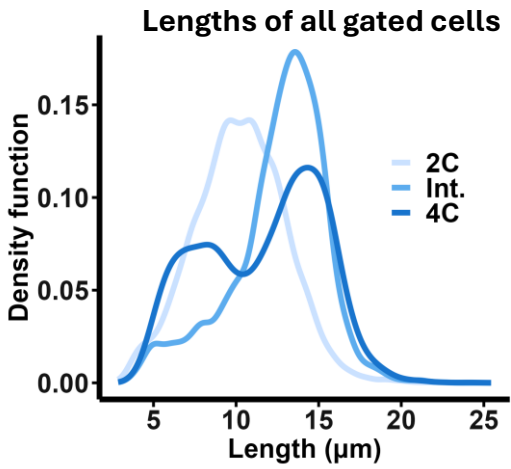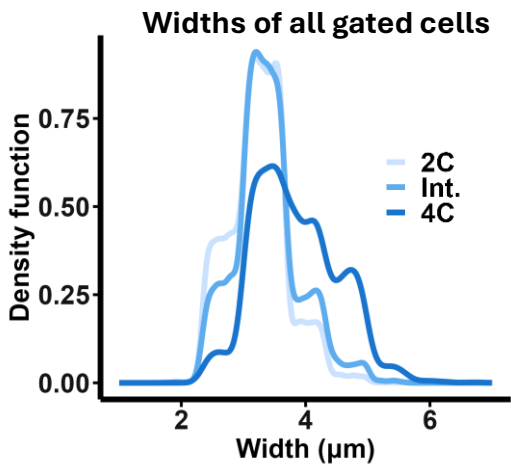

**S6 Fig**

Supplement: S6 Fig — (A) Length and (B) width distributions of C9T7 cells from the DNA intensity plot presented in Fig 3A. Top panels: cells within the 2C gate; middle panels: cells within the intermediate (2C-4C) gate; bottom: overlay of cells in all gates. (PDF) [file pone.0311367.s006.pdf]

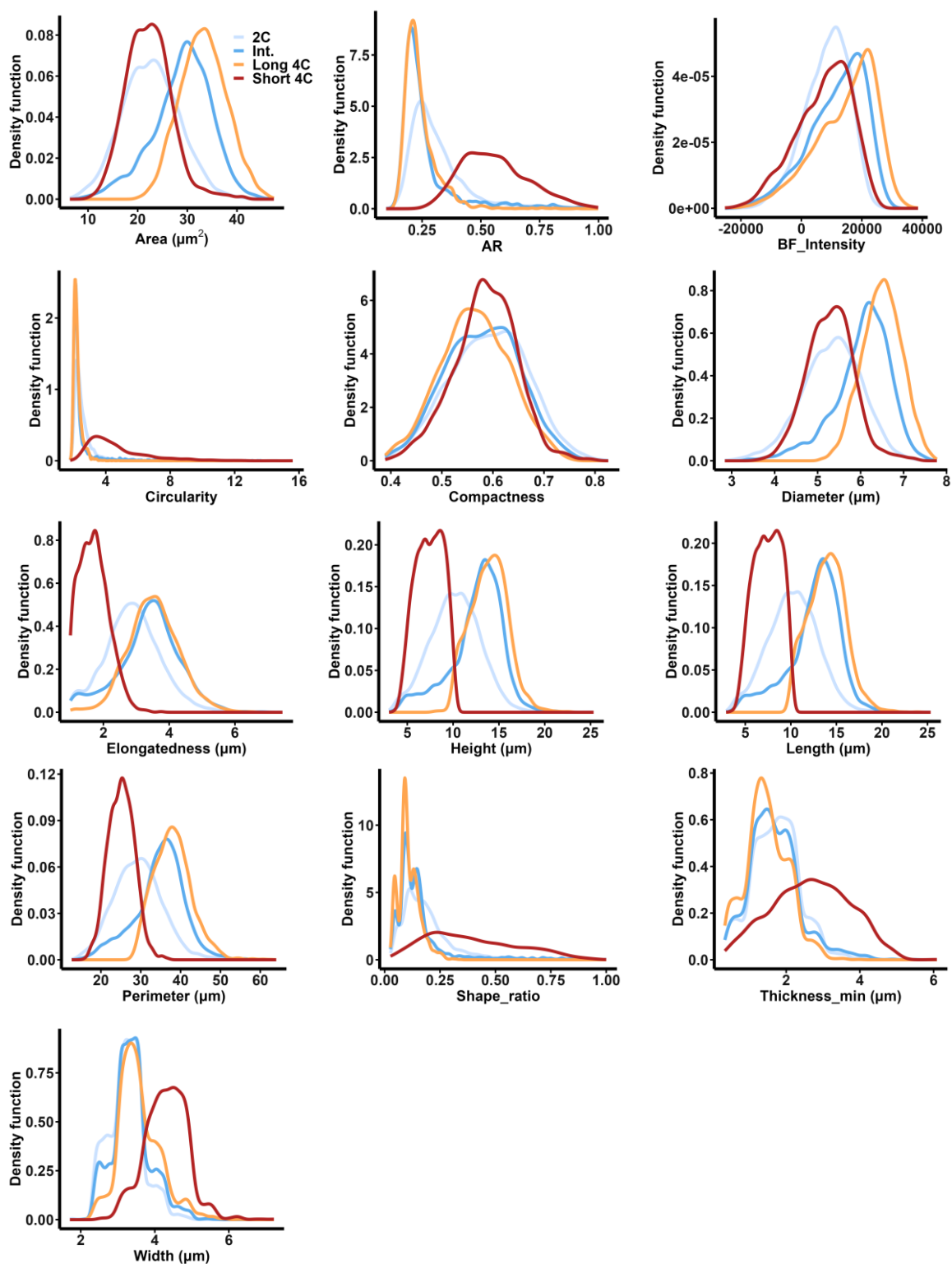

**S7 Fig**

Supplement: S7 Fig — L. mexicana C9T7 promastigotes were stained with DCO, analysed by IFC and gated according to their DNA intensity (2C, intermediate DNA content (Int.; 2C-4C) and 4C) (Fig 3A). 4C gated cells were then further gated according to their length (short: <10 μm; long ≥ 10 μm) (Fig 3B). The area, aspect ratio (AR), brightfield intensity (BF_Intensity), circularity, compactness, diameter, elongatedness, height, length, perimeter, shape ratio, thickness_min and width of the cells in each gate were then plotted, as indicated in the figure. (PDF) [file pone.0311367.s007.pdf]

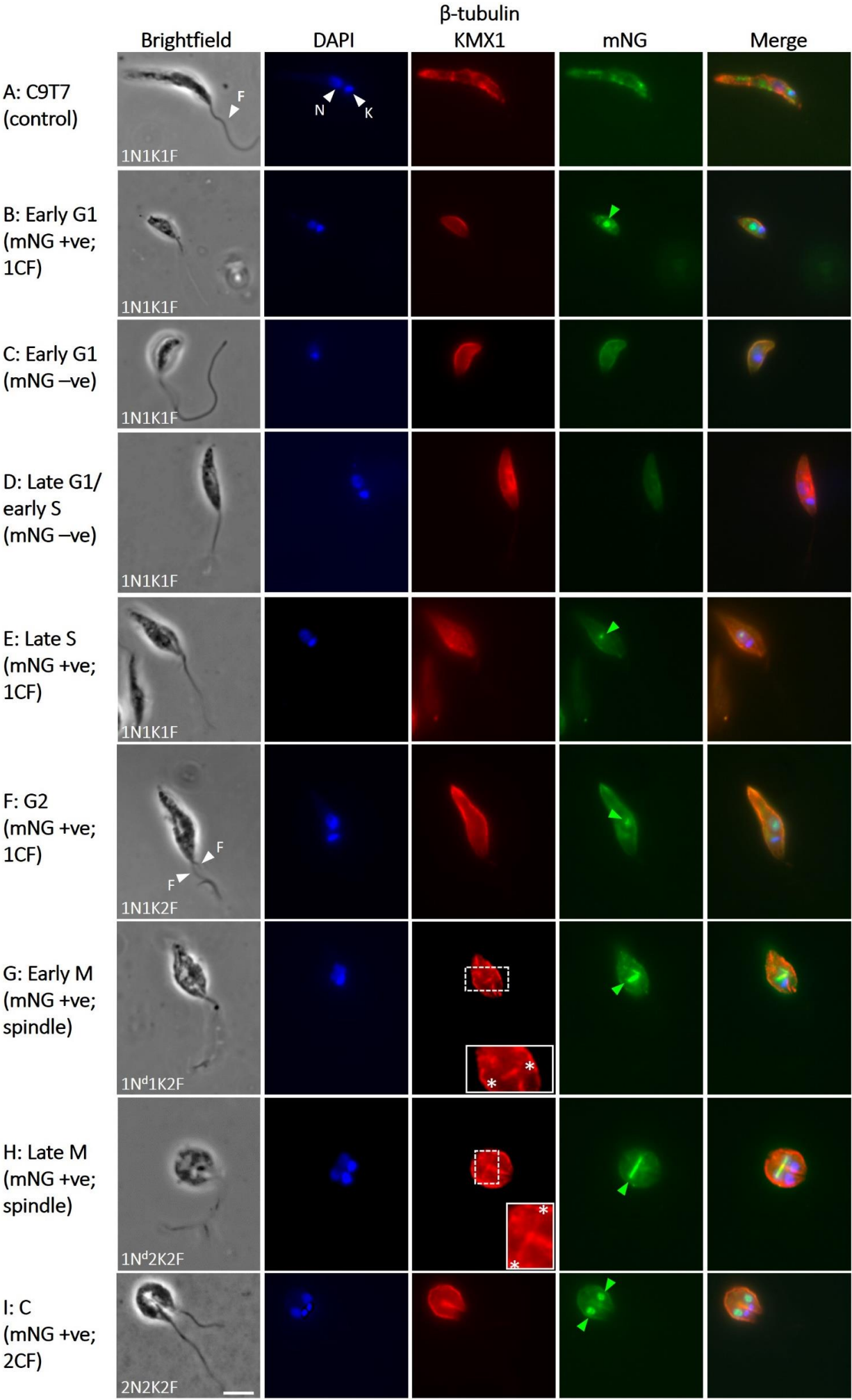

S8 Fig

Supplement: S8 Fig — Immunofluorescence was performed on L. mexicana promastigote C9T7 (control) (A) or C9T7 mNG:KINF cells (B-I) with the KMX-1 antibody to detect β-tubulin and an anti-mNG antibody to detect mNG:KINF. Cells were also stained with DAPI to visualise DNA. From left to right: brightfield, DAPI (blue), KMX-1 (red), mNG (green) and DAPI/KMX-1/mNG merged images. The number of nuclei (N), kinetoplasts (K) and flagella (F) per cell is indicated (Nd indicates a dividing nucleus), along with the cell cycle stage and mNG:KINF fluorescence pattern. White arrowheads indicate N, K and F. Dotted boxes highlight the mitotic spindle as stained by the KMX-1 antibody, which is enlarged and indicated by asterisks in the solid white line boxes; green arrowheads indicate mNG:KINF staining within the nucleus. Scale bar = 5 μm. (PDF) [file pone.0311367.s008.pdf]

**A**

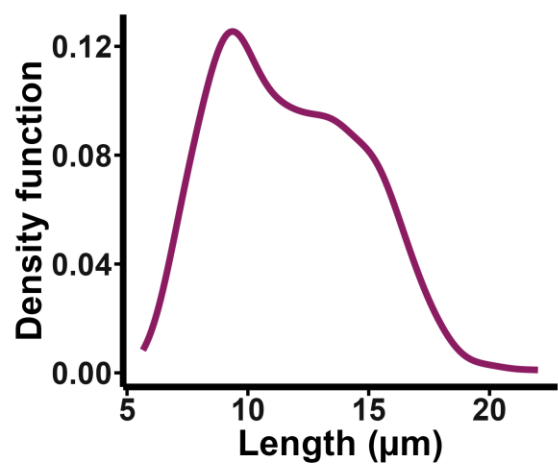

**B**

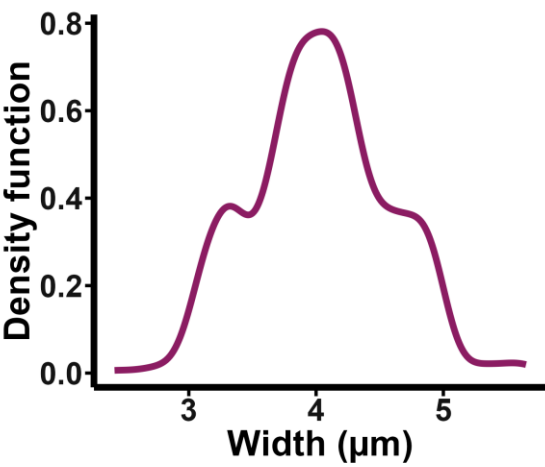

**S9 Fig**

Supplement: S9 Fig — The lengths (A) and widths (B) of C9T7 mNG:KINF cells identified as being in G2 phase were plotted. (PDF) [file pone.0311367.s009.pdf]

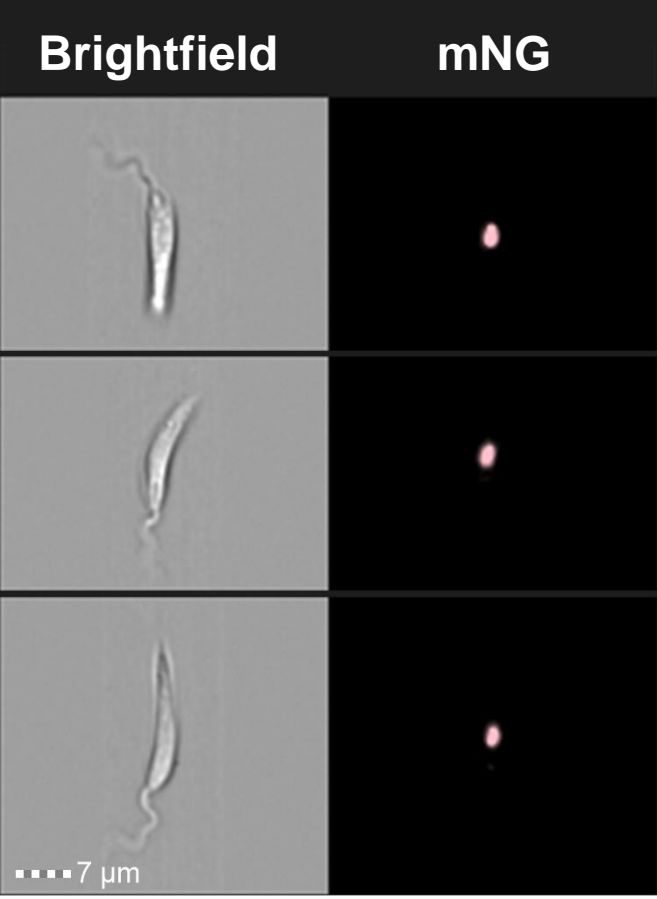

**S10 Fig**

Supplement: S10 Fig — Some long, narrow C9T7 mNG:KINF cells with a single flagellum were observed to possess a single elongated focus of mNG:KINF fluorescence that led to them being classified automatically as having a mitotic spindle. Cells such as these were manually removed from the ‘mNG:KINF spindle’ population and added to the S phase 1CF population. (PDF) [file pone.0311367.s010.pdf]

Ai

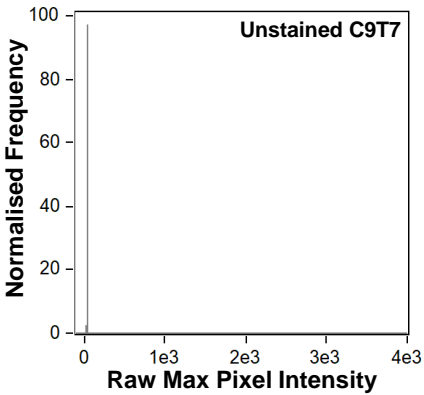

ii

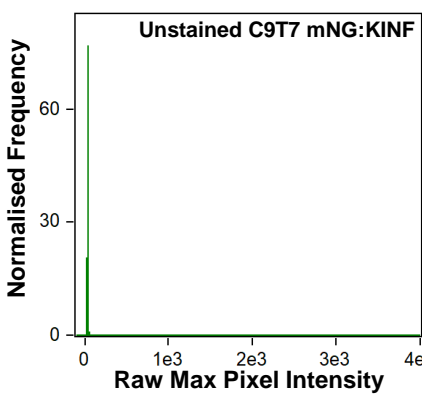

iii

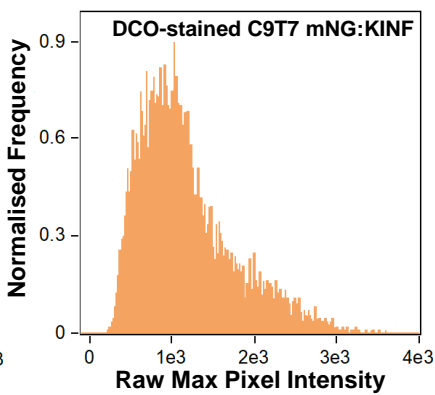

B

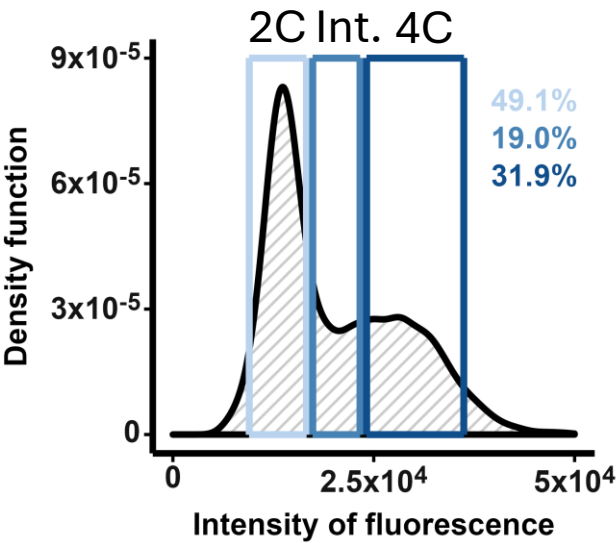

S11 Fig

Supplement: S11 Fig — C9T7 mNG:KINF cells were stained with DCO before being imaged with IFC using a 488 nm laser with a power of 3 mW to excite only DCO and not mNG:KINF to detectable levels. Fluorescence emitting in channel 3 (560–595 nm) was measured. (A) Raw max pixel intensity plots (the fluorescence intensity of the brightest pixel in each image) of (i) unstained C9T7, (ii) unstained C9T7 mNG:KINF and (iii) DCO-stained C9T7 mNG:KINF cells. (B) Example DCO fluorescence intensity plot of C9T7 mNG:KINF cells stained with DCO. 2C, intermediate (2C-4C) and 4C gates were placed as indicated (blue boxes) with the % cells within each of the gates indicated on the right. Data was from the Run 2 replicate; data from the Runs 1 and 3 replicates is in S1G Table. (PDF) [file pone.0311367.s011.pdf]

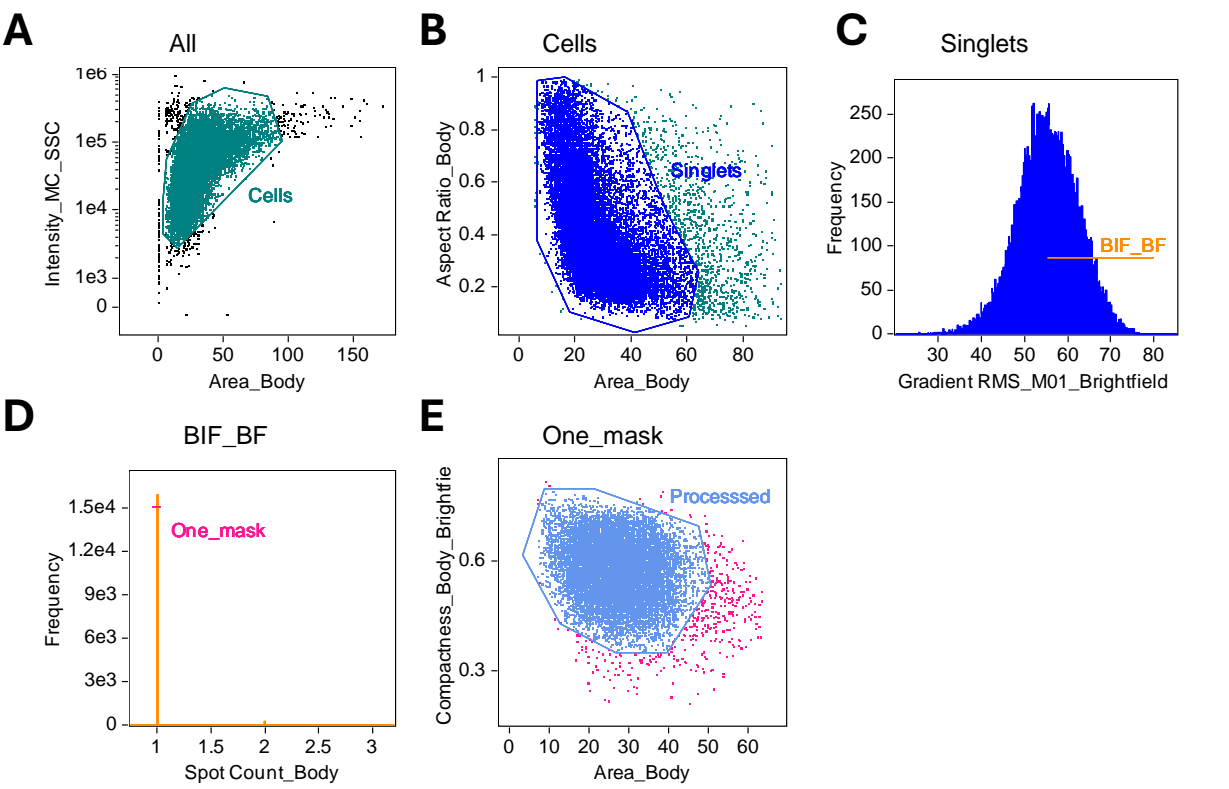

S12 Fig

Supplement: S12 Fig — Following ImageStream acquisition, data were processed using a user-defined gating strategy in IDEAS to identify single cells and gate out doublets, debris and images with artefacts. First, cell body area was plotted against side scatter, with gating applied to exclude debris and speed beads (A; teal gate). Next, singlet cells were selected using a cell body area vs aspect ratio plot (B; blue gate). The best in focus cells in bright field (BIF_BF) were identified via the Gradient_RMS parameter (C; orange bar). Images with a single mask (i.e. one object per image) were selected using “Spot Count” (D) and plotting cell body area against cell compactness enabled the removal of overlapping cells, resulting in the ‘Processed’ population (E, light blue gate), which was then utilised for morphological and fluorescence analysis. n = 30,000 C9T7 cells. (PDF) [file pone.0311367.s012.pdf]

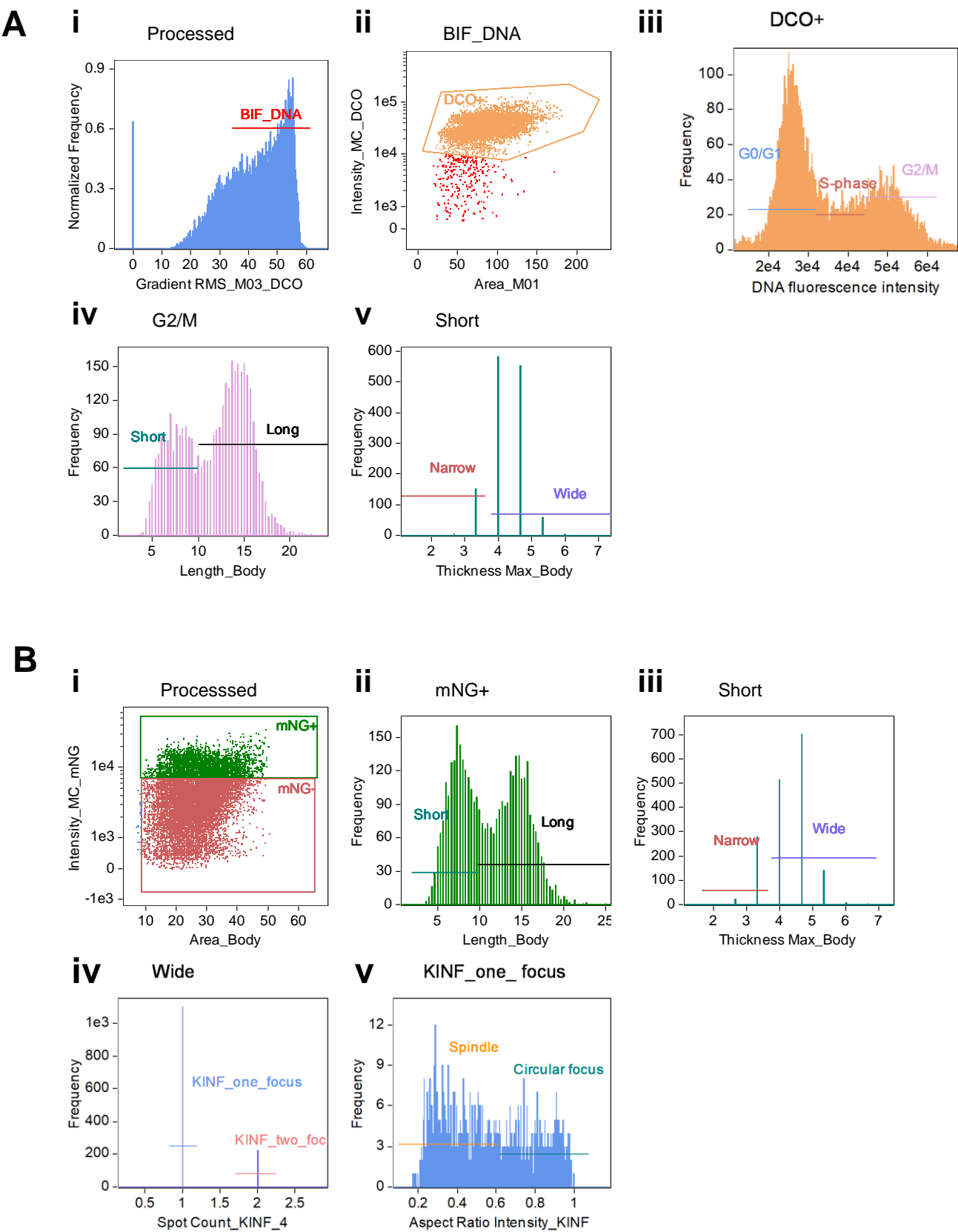

**S13 Fig**

Supplement: S13 Fig — Using a ‘processed’ population (S11 Fig), separate gating strategies were employed for the cell cycle analysis of DCO-stained C9T7 mNG:KINF cells excited with a laser power of 3 mW (A) and unstained C9T7 mNG:KINF cells excited with a laser power of 120 mW (B). (A) The best in-focus DNA images were selected using Gradient_RMS (i; red bar) and cells sufficiently stained with DCO were identified via their fluorescent intensity (ii; orange gate). The cell cycle stages of the cells were then determined based on their DCO intensity (iii; G1, S and G2/M bars). An equivalent gating strategy was employed for cells stained with other dyes (Hoechst, DRAQ5, DCV and DCR). To facilitate further resolution of cell cycle stage within the G2/M cell population of DCO-stained cells, morphological characteristics were analysed. Cell body length (iv) and width (v; using the Thickness Max parameter) were plotted to identify short (length ≤10 μm), long (length >10 μm), narrow (width <4 μm) and wide (width ≥4 μm) cells. (B) Cells expressing mNG:KINF were identified by gating on green fluorescence greater than that of the parental untagged cell line (i; green gate). Cell cycle stages were determined from the cell length (ii, short and long bars, as in (A)) and width (iii; Thickness Max, narrow and wide bars, as in (A)) and by their mNG:KINF fluorescence pattern. mNG:KINF foci were identified using masks, allowing the separation of cells with one or two mNG:KINF foci (iv). The aspect ratio intensity of cells with a single mNG:KINF focus allowed cells with a circular focus or an elongated spindle-shaped focus to be distinguished (v). (PDF) [file pone.0311367.s013.pdf]
