# Supplementary material for: Analysis of the Leishmania mexicana promastigote cell cycle using imaging flow cytometry provides new insights into cell cycle flexibility and events of short duration
Source: PLoS One. 2024 Oct 3;19(10):e0311367. doi: 10.1371/journal.pone.0311367 (PMC11449296; doi:10.1371/journal.pone.0311367)
Supplement: S14 Fig — Example images of C9T7 mNG:KINF cells that were discarded from further analysis. (A) short, wide cells with mNG fluorescence below the threshold. The majority of these cells had aberrant morphology that meant they could not be assigned to any given cell cycle stage. (B) cells assigned by IDEASTM as long and wide with two circular mNG:KINF foci. The majority of images from this group were found to be of two individual cells in close proximity that had been misclassified. (C) short narrow cells that were classified as having two circular mNG:KINF foci by IDEASTM. All of these cells were found to have unequally sized foci, suggesting that there was some additional fluorescence in the region of the kinetoplast (white arrowheads). (PDF) [file pone.0311367.s014.pdf]

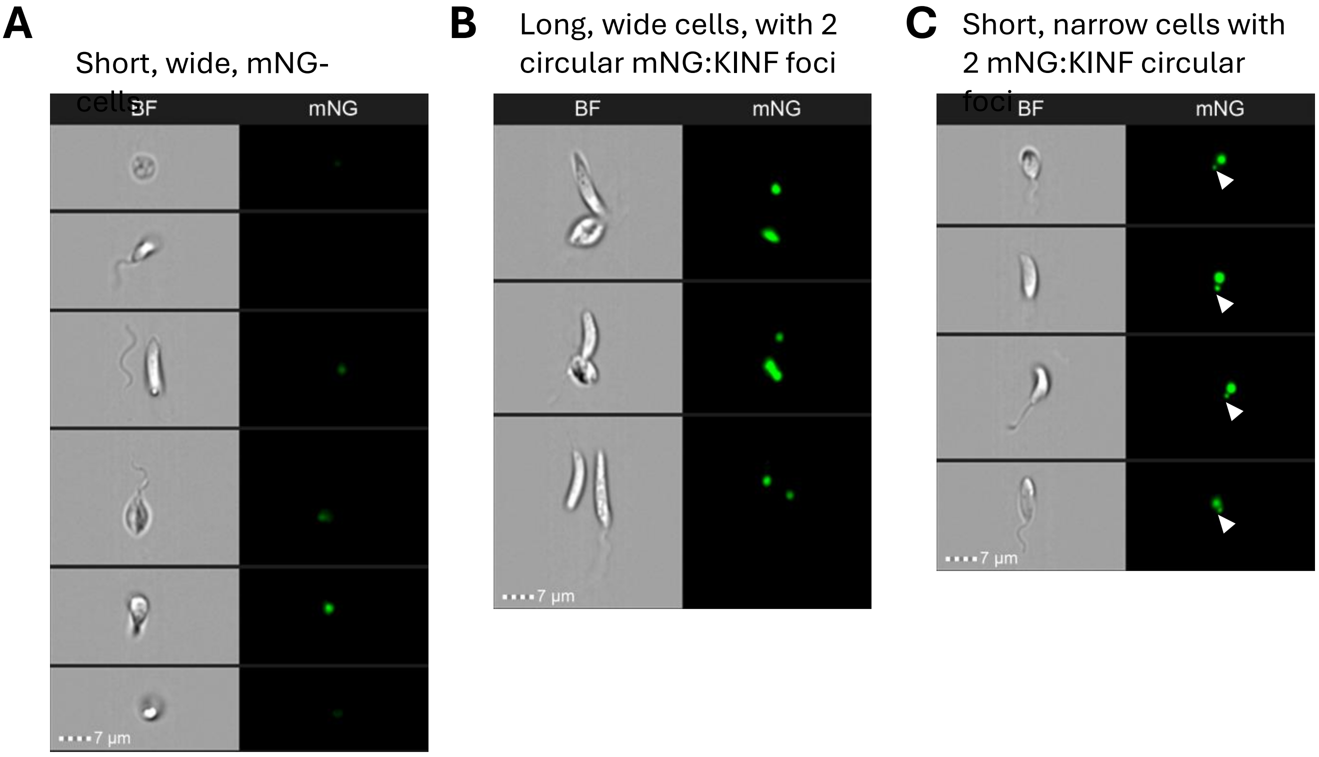
A
Short, wide, mNG- cells
BF
mNG
7 µm
B
Long, wide cells, with 2 circular mNG:KINF foci
BF
mNG
7 µm
C
Short, narrow cells with 2 mNG:KINF circular foci
BF
mNG
7 µm

S14 Fig
