# Supplementary material for: Analysis of the Leishmania mexicana promastigote cell cycle using imaging flow cytometry provides new insights into cell cycle flexibility and events of short duration
Source: PLoS One. 2024 Oct 3;19(10):e0311367. doi: 10.1371/journal.pone.0311367 (PMC11449296; doi:10.1371/journal.pone.0311367)
Supplement: Attachment — Submitted filename: Response to reviewers comments_final.pdf [file pone.0311367.s016.pdf]

Response to reviewers' comments – Howell et al Manuscript number: PONE-D-24-20727

We would like to thank the two reviewers and editor for their thorough and helpful reviews of our manuscript. We have detailed our responses to their comments below.

>This manuscript presents a state-of-the art, IFC-based pipeline to combine the population-level and quantitative analysis advantages of flow cytometry to bring to bear on analyses of the Leishmania cell cycle in live cells. One advantage of this high-throughput approach, in principle, is to allow capture of hard-to-identify populations of cells transiting through short-lived sectors of the cell-cycle. The authors claim several novel observations: including a newly detailed description of the Leishmania promastigote cell cycle and establishing the durations of distinct cell cycle phases. The authors also present a potentially useful methodological proof of principle for IFC in evaluation of the action of anti-Leishmania drug candidates, as they may relate to the cell cycle. There appear, however, to be a several caveats in their approach and findings (see reviewer comments), that need to be addressed.

The authors must please respond point-by-point to each of the reviewers' comments. For clarification, **they do need to respond to Reviewer 2's concerns regarding the anti-tubulin staining.**

>The authors used the terms "elongatedness" and "length" (e.g. line 220); it is unclear whether these terms are referring to the same or different parameters.

These are distinct parameters. Elongatedness is height/width and is defined in the methods (lines 885-886). However, to aid clarity, we have added this definition at first use to the main text (line 233).

>For reviewer 2, please also clarify whether the cell cycle analyses in Figs 2 and 3 were performed on mNG-KINF-expressing cells, or not .

As already stated in the legends to these figures, these data were from analyses performed with C9T7 cells (ie parental not mNG-KINF). We have added 'C9T7' to the description in the Fig 2B legend (line 200) to clarify that these analyses were of the C9T7 cells presented in Fig 2A images. We have also added 'C9T7' references in the main text description (lines 149 and 226) to avoid confusion.

Some minor issues with language and typos were also noted:

e.g.s

Line 17: "Promastigote Leishmania mexicana have…"

This is intended as a plural reference to all *Leishmania mexicana* promastigote parasites and is therefore correct as it stands.

Line 371: "Visually inspected for the presence of two visible flagella" (remove "Visually" or "visible").

We have swapped 'visually inspected' for 'inspected by eye'. It is important to highlight that this analysis was not done by computer, and to keep the 'visible' flagella description – this refers to flagella that have emerged from the flagellar pocket and can be seen by light microscopy – some cells will have internal flagella that have started to form but haven't yet emerged from the cell and will therefore not be visible by eye (now line 377).
================================

Please submit your revised manuscript by Aug 02 2024 11:59PM. If you will need more time than this to complete your revisions, please reply to this message or contact the journal office at plosone@plos.org. When you're ready to submit your revision, log on to https://www.editorialmanager.com/pone/ and select the 'Submissions Needing Revision' folder to locate your manuscript file.

Please include the following items when submitting your revised manuscript:

- A rebuttal letter that responds to each point raised by the academic editor and reviewer(s). You should upload this letter as a separate file labeled 'Response to Reviewers'.
- A marked-up copy of your manuscript that highlights changes made to the original version. You should upload this as a separate file labeled 'Revised Manuscript with Track Changes'.
- An unmarked version of your revised paper without tracked changes. You should upload this as a separate file labeled 'Manuscript'.

If you would like to make changes to your financial disclosure, please include your updated statement in your cover letter. Guidelines for resubmitting your figure files are available below the reviewer comments at the end of this letter.

If applicable, we recommend that you deposit your laboratory protocols in protocols.io to enhance the reproducibility of your results. Protocols.io assigns your protocol its own identifier (DOI) so that it can be cited independently in the future. For instructions see: https://journals.plos.org/plosone/s/submission-guidelines#loc-laboratory-protocols. Additionally, PLOS ONE offers an option for publishing peer-reviewed Lab Protocol articles, which describe protocols hosted on protocols.io. Read more information on sharing protocols at https://plos.org/protocols?utm_medium=editorial-email&utm_source=authorletters&utm_campaign=protocols.

We look forward to receiving your revised manuscript.

Kind regards,

Ben L. Kelly, Ph.D.
Academic Editor
PLOS ONE

Journal Requirements:

When submitting your revision, we need you to address these additional requirements.

1. Please ensure that your manuscript meets PLOS ONE's style requirements, including those for file naming. The PLOS ONE style templates can be found at https://journals.plos.org/plosone/s/file?id=wjVg/PLOSOne_formatting_sample_main_body.pdf and https://journals.plos.org/plosone/s/file?id=ba62/PLOSOne_formatting_sample_title_authors_affiliations.pdf

2. Thank you for stating in your Funding Statement:
"This work was supported by the Engineering and Physical Sciences Research Council (PhD studentship no. EP/R513222/1 held by J.H.), the Royal Academy of Engineering (Research Fellowship no. RF/201718/1741 awarded to M.J.). and the Cunningham Trust (PhD studentship grant (PhD-CT-19-14) awarded to T.H. and M.J., and held by S.O.)."

Please provide an amended statement that declares *all* the funding or sources of support (whether external or internal to your organization) received during this study, as detailed online in our guide for authors at http://journals.plos.org/plosone/s/submit-now. Please also include the statement "There was no additional external funding received for this study." in your updated Funding Statement.
Please include your amended Funding Statement within your cover letter. We will change the online submission form on your behalf.

Done.

3. Thank you for stating the following in the Acknowledgments Section of your manuscript:
"The authors would like to thank Pj Chana from Cytek Bio for useful advice, discussions and training on IFC. This work was supported by the Engineering and Physical Sciences Research Council (PhD studentship no. EP/R513222/1 held by J.H.), the Royal Academy of Engineering (Research Fellowship no. RF/201718/1741 awarded to M.J.). and the Cunningham Trust (PhD studentship grant (PhD-CT-19-14) awarded to T.H. and M.J., and held by S.O.)."

We note that you have provided funding information that is currently declared in your Funding Statement. However, funding information should not appear in the Acknowledgments section or other areas of your manuscript. We will only publish funding information present in the Funding Statement section of the online submission form.
Please remove any funding-related text from the manuscript and let us know how you would like to update your Funding Statement. Currently, your Funding Statement reads

as follows:
"This work was supported by the Engineering and Physical Sciences Research Council (PhD studentship no. EP/R513222/1 held by J.H.), the Royal Academy of Engineering (Research Fellowship no. RF/201718/1741 awarded to M.J.). and the Cunningham Trust (PhD studentship grant (PhD-CT-19-14) awarded to T.H. and M.J., and held by S.O.)."

Please include your amended statements within your cover letter; we will change the online submission form on your behalf.

[Note: HTML markup is below. Please do not edit.]

Done.

Reviewers' comments:

Reviewer's Responses to Questions

Comments to the Author

1. Is the manuscript technically sound, and do the data support the conclusions?

The manuscript must describe a technically sound piece of scientific research with data that supports the conclusions. Experiments must have been conducted rigorously, with appropriate controls, replication, and sample sizes. The conclusions must be drawn appropriately based on the data presented.

Reviewer #1: Yes

Reviewer #2: Partly

---

2. Has the statistical analysis been performed appropriately and rigorously?

Reviewer #1: Yes

Reviewer #2: Yes

---

3. Have the authors made all data underlying the findings in their manuscript fully available?

The PLOS Data policy requires authors to make all data underlying the findings described in their manuscript fully available without restriction, with rare exception (please refer to the Data Availability Statement in the manuscript PDF file). The data should be provided as part of the manuscript or its supporting information, or deposited

to a public repository. For example, in addition to summary statistics, the data points behind means, medians and variance measures should be available. If there are restrictions on publicly sharing data—e.g. participant privacy or use of data from a third party—those must be specified.

Reviewer #1: Yes

Reviewer #2: No

---

4. Is the manuscript presented in an intelligible fashion and written in standard English?

PLOS ONE does not copyedit accepted manuscripts, so the language in submitted articles must be clear, correct, and unambiguous. Any typographical or grammatical errors should be corrected at revision, so please note any specific errors here.

Reviewer #1: Yes

Reviewer #2: Yes

---

5. Review Comments to the Author

Please use the space provided to explain your answers to the questions above. You may also include additional comments for the author, including concerns about dual publication, research ethics, or publication ethics. (Please upload your review as an attachment if it exceeds 20,000 characters)

Reviewer #1: The paper by Howell et al details a new method for analyzing the cell cycle in L. Mexicana using imaging flow cytometry. The paper is an important contribution because analyzing cell cycle using conventional microscopy methods is very labor intensive and looking for rare phenotypes can be challenging with this technique. While conventional DNA staining can separate cells into G1, S, and G2, the addition of images combined with the flow cytometry technique allows further delineation for stages of the cell cycle based on length and width measurements along with other morphological characteristics so that early and late G1 phase cells, S phase, and G2/M/cytokinesis cells can be distinguished. In addition, using a neon green KINF reporter cell line can help to distinguish cells in the mitotic stage from G2 cells. Imaging flow cytometry allows for much higher throughput and less labor intensive cell cycle analysis. These methods will be especially useful when analyzing the effects of various drugs or other genetic manipulations within the L. Mexicana system. Using a combination of IFC techniques, the authors were able to come up with detailed time estimates for how L. mexicana progresses through the different stages of the cell cycle. The paper is well written and clear, and most of my comments are minor suggestions. While I plan to enumerate a few suggestions in the ‘major’ category, the authors should not feel bound

to follow them; in my opinion the paper is publishable pretty much as is.

We thank the reviewer for their positive review of our manuscript.

Major points

• I'm not entirely sure that the data presented in Figure 4 is necessary. There is quite a bit of background staining here for the neon green, as shown in the control cells that do not have the construct, while the IFC imaging looks comparatively much cleaner. While the authors use a beta-tubulin stain for these conventional microscopy images, this stain doesn't feature much at all within the text and doesn't seem necessary for making any of their main points. The spindle staining shown in Figure 4H and the double stain shown in Figure 4I are clearly visible in Figure 5A. The lack of KINF staining in G1/S cells is also more clearly seen in the IFC analysis because there is less background. For this reason, Figure 4 doesn't seem to contribute much to the big picture.

We agree that the β-tubulin immunofluorescent staining in the fixed cells is not as high quality as the live cell fluorescence imaging by IFC, but we believe it is important to include as a control to demonstrate that the mNG:KINF signal colocalises with the mitotic spindle, as expected. Unfortunately, Fig 5A doesn't show this colocalisation – the two panels in this figure show the mNG:KINF signal and the automatic mask applied to it, rather than a β-tubulin signal, so don't provide this necessary control. We tried hard to optimise the conditions for the anti-mNG staining but without success. However, we only use this to show that there is mNG:KINF signal at the spindle, and the rest of our analysis of mNG:KINF localisation was performed with live cells and IFC, so do not believe this detracts from our interpretation of mNG:KINF cell cycle distribution. In view of the reviewers' comments about this figure, we have moved it to the Supplementary data (S8 Fig) so that it doesn't detract from the main story of the manuscript.

• Figure 8 presents some nice data on a drug previously shown to alter the cell cycle. The authors show an arrest at the 4C stage using conventional PI staining, and then further analyze the cells using the KINF neon green marker to show that the cells get stuck before mitosis (if I'm interpreting this right). I found myself wondering how the conventional PI stain for drug treated cells compares to the dicycle orange stain that the authors so nicely showed earlier. I realize that the dicycle orange stain is not compatible with the neon green marker, so it might be necessary to get additional data to show how these two analyses compare. While I don't think this is absolutely necessary, it might be a nice addition to the paper if it is not too labor intensive. It would help to show that the dicycle orange analysis can still be useful for analyzing drug-related cell cycle defects in Leishmania lines that do not carry the KINF marker, and that it's comparable to the PI analysis that everyone is very familiar with in cases where the cell cycle is perturbed.

Given that DCO quantitation of DNA in live cells is equivalent to PI quantitation of DNA in fixed cells (Fig 2B-D and S2A,B Fig), we see no reason that DCO staining on its own could not be used for investigating cell cycle defects following drug treatment. Our prediction is that this would allow changes to the 2C, intermediate and 4C DNA fluorescence peaks, or the appearance of abnormal peaks (eg 8C for polyploid cells) to be identified, in the same manner as PI does in fixed cells, although testing this was not possible within the time-constraints of this project (the first two authors are now writing up their PhDs). Additionally, the ability to combine DCO staining of live cells with analysis of cell morphology should allow greater resolution of some cell cycle stages over PI (e.g. compare DCO staining alone (equivalent to PI) and DCO staining + cell morphology in new summary Table 2 – see below). However, like PI, without employing KINF fluorescence, DCO staining alone would not provide any information on mitotic spindle formation, and even if combined with morphological analysis, could not distinguish G2 cells from M phase cells. We therefore hope that in future a different fluorescent marker compatible with DCO could be employed to tag KINF to enable simultaneous detection.

• The authors present two main techniques for analyzing the cell cycle with IFC, one with dyecycle orange and one with the KINF marker. If I understand the data correctly, these techniques can't be performed at the same time because of spectral overlap. Given that this is the case, it might be nice for the authors to help guide readers as to which technique is the most useful for a particular experimental study. For example, those interested in characterizing defects in G2/M might find the KINF staining more useful, whereas people interested in distinguishing problems entering S phase might be able to rely on the length measurements combined with the dicycle orange stain.

The techniques can't be performed simultaneously because they require different excitation laser powers for optimal staining rather than because of spectral overlap. However, in line with the reviewer's suggestion, a new summary table (Table 2) and associated text (see lines 631-646) has been added to the manuscript that summarises the capabilities of DCO staining vs mNG:KINF fluorescence alongside morphological analysis.

Minor points

• In Figure 2 the figure legend makes the point that the nucleus divides first, that point should be made in the main text

This clarification has been added (lines 186-187), as requested.

• Figure 3B, it would be helpful to put in a title that marks these cells specifically in the 4C gate

We have added a key for the 4C cells, and also updated the colours of Fig 3A so that the 4C gate matches the colour of the curve in Fig 3B.

• Figure 4, label the stain as beta-tubulin above KMX1 antibody label

This has been added (now S8 Fig).

• Should 4D also say mNG-ve? Stay consistent with these labels

The mNG+ve vs mNG-ve labels were used simply to distinguish the two early G1 populations (which are virtually identical in morphology). However, in view of the reviewer's comment, we have added mNG staining descriptions to all panels (now S8 Fig).

• In Figure 4, it would be nice if G, and H also had green arrowheads to indicate positive staining to be consistent

These have been added (now S8 Fig).

• Figure 6 it would be helpful to put a legend in a corner indicating what arrows and stars mean so that readers don't have to go hunting in the legend

A key has been added to this figure (now Fig 5).

• It would be helpful to summarize the specific contribution of the neon green at the end of that section, it lets us distinguish between this and that

We have added the sentence 'mNG:KINF staining therefore provides additional resolution of cell cycle progression compared to DNA staining alone, allowing the unambiguous identification of cells with a mitotic spindle as well as post-mitotic cells and cells in early G1.' to the end of this section (lines 317-320).

• Figure 8 please put full name of drug in figure

This has been done (now Fig 7).

Reviewer #2: This manuscript describes a new cell cycle analysis pipeline for live Leishmania mexicana that utilizes state of the art flow cytometry that is directly coupled to both phase contrast microscopy and fluorescence intensity microscopy. This allows very rapid analysis of large numbers of live cells on the basis of three different imaging modalities thus providing a large saving in time and labor. The authors argue that this type of analysis can reduce artifacts while greatly increasing the efficiency of experiments designed to analyze mutant or drug-induced perturbations in the L. mexicana cell cycle.

While the data and procedures that are the primary focus of this manuscript are more suitable to a protocol manuscript, this submission is as a field research report. Unlike a typical field research report, this manuscript does not present a testable biological hypothesis or propose to test an important new finding using new methodology, but rather purports to discover and list technical advantages of a cutting edge flow cytometry instrument.
In this sense, this report is one-sided in primarily only providing evidence in support of the protocols used in this approach and does not discuss or provide balanced insight to the apparent weaknesses and compromises inherent in the protocols.

We thank the reviewer for their comments and have taken them on board. We have now mentioned some disadvantages of IFC (lower magnification/resolution and the need to remove out-of-focus cells by gating), in the discussion (lines 587-591). However, we did not find these disadvantages to be limiting for the study we carried out. (Please see below for a discussion of mCherry fluorescence). Because of the new insights into *Leishmania* cell cycle biology, we strongly believe this manuscript warrants reporting as a research paper rather than a protocol.

Furthermore, this report documents details of protocol outcomes that are purported to be important first-of-their kind conclusions.
1) This report is not the first imaging flow cytometry analysis of Leishmania. Nor the first to make correlations of cell cycle state to morphology albeit with previously published reports that did not utilize IFC.

We agree with the reviewer and indeed, our submitted manuscript included many references to previous IFC and morphological studies in Leishmania throughout (refs 3-10, 37-42, 47-48, 55, 57, 68-69 and 75-80). While we outline the new insights into the cell cycle that we have gained from using IFC, we only claim 'firsts' for a) being able to identify G2 cells and new G1 cells and b) determination of the individual timings of G2, mitosis and post-mitosis phases (lines 25-34 and 674-676), which we believe are justified.

2) Necessity of using very high laser power (120mW vs 3mW for DNA stains) to detect mNeon GFP and inability to detect mCherry fusions at all. This is a major limitation of this instrument which should be discussed and experimentally assessed as many investigators rely on GFP/RFP-based fluorescence in live cell analyses. It seems that IFT can detect broad or very bright Leishmania fluorescence, it is not capable or barely capable of detecting organellar fluorescence. Had the authors succeeded in finding a two-channel solution, perhaps mScarlet3 fusions, to use with mNeon, this report would have been more compelling.

In our opinion, 120 mW is not a very high laser power. It is well within the normal range of the imaging flow cytometer (the top 488 laser power being 200 mW). Checking the literature, we find that other researchers have used a range of laser

powers for the 488 laser from e.g. 25 mW (https://doi.org/10.1038/ncomms10256) to 200 mW (https://doi.org/10.1016/j.ymeth.2016.07.002) for different fluorophores, so our 120 mW sits in the middle of this range. Thus, we strongly believe the reason we had to use 120 mW rather than 3 mW to detect mNeonGreen is a limitation of the tag rather than the imaging technology – mNG:KINF also requires longer exposure times and is more labile than dyes such as DCO and DAPI when using standard fluorescence microscopy to detect it.

Similarly, while we found that our mCherry:KINF fusion protein could not be detected by IFC, it was also faint when imaging by standard fluorescence microscopy, and thus we think it is more likely to be a property inherent to this particular fusion than a general issue with using mCherry. We did not investigate other mCherry fusions Therefore, we cannot safely conclude that IFC is unsuitable for detecting all mCherry fusions, or that it is barely capable of detecting organellar fluorescence based on this single incidence; this would require numerous other cell lines expressing fusion proteins of differing known abundances to be generated and tested, and that is beyond the scope of this work to investigate.

We agree that it would have been ideal to have found a two-channel solution, but that unfortunately wasn't possible within the timeframe of the first two authors' PhD projects. Generating fluorescently tagged *Leishmania* requires the generation of bespoke plasmids, sometimes with codon-optimised coding sequences to achieve best expression; we had suitable plasmids available for mNG and mCherry, but would have needed to generate additional plasmids to test other fluorescent reporters.

In view of the reviewer's comments, we have edited the discussion of mCherry fluorescence (lines 660-672) to make it clearer that we have not ruled out using mCherry for other fusion proteins, and have cited a paper where mCherry has been used successfully in *Toxoplasma gondii* for IFC (https://doi.org/10.1128/iai.01643-14)

3) Lack of dose response data to justify the stated DNA probe concentrations prior to IFC. Raw DCO data are not included in Figure S2. DCO data presented in Figures 2 and 3 appear to have undergone further gating and filtering to enrich for high-staining cells; a procedure not utilized for the other DNA stains. The conclusion that the other stains are not quantitative is not based on thorough experimental evidence.

We did test additional concentrations of the various DNA probes (including DCO), but didn't put these in our original manuscript as we didn't feel they would add much to the story – these dyes were just included as a brief backstory to explain why we chose DCO, and to save other *Leishmania* researchers wasted effort. However, in view of the reviewer's comments, these are now included in S2C-G Fig, and in the methods (lines 813-817). The accompanying text (lines 173-194) has now been updated to describe these additional data. We have also made it clearer in

the results that these dyes were not quantitative *under the conditions tested* (see lines 174-182). We had already stated this in the discussion (lines 605-617).

The reviewer is correct in surmising that, unlike DCO-stained cells, cells stained with the alternative DNA dyes that were tested, while gated according to the pipeline presented in S12 Fig, were not gated to identify high staining cells (pipeline in Fig S13Ai-iii). The extra gating that was performed on DCO cells (Fig S13Ai-iii) was undertaken to permit further morphological analysis, which required rigorous gating to identify a robust population to work from. However, the referee's point is valid and to keep the data consistent, we have now performed equivalent gating for the alternative DNA dyes (data presented in S2C-F Fig, and stated this in the methods (line 848) and Fig S13 figure legend (lines 1286-1289).

4) Cell cycle analysis using DCO (Figures 2 and 3; DCO+ in Fig S12) were performed on mNG transfected cells. It is argued that although DCO and mNG share overlapping excitation/emission profiles, that mNG did not contribute to these data. Given that there is stated variability in mNG expression levels and variability in DCO staining strength (which required gating and filtering), it remains distinctly possible that mNG materially contributed to these spectra (especially the lower intensity data). This experiment should be performed on cells without mNG.

Fig 2 and 3 were not performed on mNG:KINF cells but on parental C9T7 cells without DCO, as was stated in the legends to these figures (see earlier response to editor). The intensity plot of DCO fluorescence in Fig S10 (now S11B Fig) shows C9T7 mNG:KINF cells stained with DCO and imaged with a laser power of 3 mW (aiming to excite only DCO and not mNG:KINF to detectable levels in Channel 3 (560-595 nm) of the ImageStream which was used to detect DCO fluorescence). This plot of DCO fluorescence provides essentially the same cell cycle profile as the Fig 3A plot for parental cells, indicating that there is no significant mNG:KINF signal excitation at 3 mW laser power that is detectable within Channel 3. We have now also added raw max pixel plots for unstained C9T7 and C9T7 mNG:KINF cells, as well as the C9T7 mNG:KINF cells stained with DCO, excited with a 488 nm laser power of 3 mW to new S11A Fig, showing that fluorescence emittance in Channel 3 is negligible in a mNG:KINF background in the absence of DCO. Thus, any mNG fluorescence (which emits maximally at 517 nm) excited by a 3 mW laser power does not contribute to the emission spectra in Channel 3 when analysing DCO fluorescence intensity.

5) DCO raw intensity curves for data in the supplementary excel table are not provided. Without the curves, the relative qualities of the peak intensity values recorded in the tables cannot readily be assessed. The curves should be in the SI.

We are not sure what the reviewer is requesting here, as we believe we have provided all raw intensity curves. The DCO intensity curves corresponding to the data in S1B and S1G Table are already provided in Fig 2 and S11 Fig, respectively.

6) The discovery of pleiotropic cleavage furrow progression is weakened by lack of live cell time-lapse corroboration with the IFC data, plus worrying artifacts of partially -of-focus cleavage furrows that populate the dataset. The limited depth of field of the microscopy reduces the confidence of the direction of furrow formation conclusions, especially since they disagree with prior published results.

These data do not disagree with previous published work – we show that many cells do indeed divide as previously reported (from the anterior to the posterior). However, we analysed far greater numbers of furrowing cells than previous studies, classifying furrows in 302 cells with mitotic spindles and 479 post-mitotic cells, compared to the analysis of fewer than 150 post-S phase cells (which would have also included G2 and early mitosis cells without furrows) in Wheeler et al, 2011 and just 4 furrowing cells in Hair et al, 2024. We believe this higher throughput enabled us to identify rare phenotypes that previous work would likely not have confidently detected within the lower number of cells analysed, and therefore this provides an extension to existing data. The demonstration that *Leishmania* is pleiomorphic for cytokinesis is not new since furrowing from each end of the parasite was previously reported in the closely related *Leishmania* species, *L. major* (https://doi.org/10.1128/ec.05118-11)

While we agree that live cell time-lapse imaging would add weight to our conclusions, this would be technically very difficult and extremely time-consuming to do, given the rarity of these events, that we are imaging in solution without any cell cycle synchroniser added and that these are motile cells. Imaging on a slide or in a matrix to isolate and contain the dividing cell of interest would add traction forces and potentially alter the results. This is therefore not within the scope of this study to perform.

However, to provide further evidence of pleiotropic cleavage furrowing in *L. mexicana*, we looked at the cell images available from the LeishTag online database https://www.leishtag.org/ . LeishTag is an ongoing project where all *L. mexicana* proteins that do not have a close orthologue in *T. brucei* (which have already been analysed by the sister project TrypTag) are being tagged with mNeonGreen at the N and/or C-terminus in order to determine their localisation. Multiple microscopic fields are captured for each tagged cell line and brightfield, DAPI, mNG & merged images uploaded to LeishTag. Scrolling through the available images revealed numerous examples of dividing cells with 2 flagella undergoing pleiotropic furrowing. In addition to cells observed in the process of dividing from anterior to posterior, multiple examples of cells dividing from the posterior, bidirectionally or initiating furrowing internally were found (see attached PDF file: LeishTag dividing cells). These examples were observed across multiple tagged cell lines, and as it is not expected, in the vast majority of cases, for the addition of

an mNG tag to influence growth or cell cycle progression, this suggests these mechanisms of cell division are naturally present in *L. mexicana* promastigote populations. Hence, these examples provide independent corroboration of our observations.

7) The DCO fluorescence intensity decay spectra in Figure S4 do not support the conclusion that there is significant decay in the peak signal over 46 minutes; peak signal remains unchanged by visual inspection (2.5 x 10fourth) but there is an obvious rightward shift of reduced intensity for the smaller second peak. This calls into question whether the second smaller peak represents nuclear DNA while the major peak represents kinetoplast DNA.

We did not mean to claim that the peak signal decays but that there is a decay in the intensity of the 4C fluorescence peak, and have now clarified this in the text (line 188). We have also added cell cycle modelling data (acquired using FCS Express) of these spectra (new S4B Fig and line 189), showing that the decay in fluorescence intensity interferes with modelling of the cell cycle stage peaks.

In the intensity spectra, each point derives from the total fluorescence of a single cell, which is a combination of DNA fluorescence from the nuclei and kinetoplasts present in that cell. Thus, there is no way the two peaks could represent separate nuclear and kinetoplast DNA intensities. We also know from visual inspection of cells in our 2C and 4C peaks that they have morphologies consistent with cells in G1/S and G2/M/C, respectively – indeed, being able to inspect the cells in each peak is one of the advantages of IFC over standard flow cytometry.

8) KMX1 tubulin staining appears very poor in Figure 4, with the mitotic spindle barely resolved from background. It could be argued that the signal to noise of the mNG KINF signal allows better visualization of the morphogenesis of the mitotic spindle. Furthermore, KINF staining within the nuclear compartment in interphase prevents a clear assessment of the G2/M transition point as the initiation of spindle morphogenesis cannot be marked; perhaps centrin or similar could be used to mark G2/M with the advent of centrosome duplication (but it is unlikely that IFC could detect centrosome staining).

We agree that mNG:KINF fluorescence provides better visualisation than KMX staining. We didn't use KMX staining to mark the initiation of spindle morphogenesis, but purely as a control to show that the mNG:KINF signal was marking the spindle. As discussed above in our response to reviewer 1's comment on this issue, we agree the IFA staining is not ideal, but we were unable to optimise these data further. We have moved old Fig 4 to supplementary data (new S8 Fig) so that it does not get in the way of our main story. With respect to other possible markers, Leishmania does not possess centrosomes, and centrin proteins are found at the basal bodies instead (https://doi.org/10.1074/jbc.M106806200; https://doi.org/10.1074/jbc.M106806200). These are not suitable markers for the

G2/M transition as the basal bodies duplicate during G1 phase, and we are not aware of any more suitable markers than KINF for the onset of spindle formation (https://doi.org/10.1016/j.molbiopara.2018.12.003).

9) Gating strategy (S11) relied on 'visual inspection' of ~100 cells corresponding to individual points... Please confirm in the methods that each replicate set relied on independently assessed visual gating.

Yes, this was the case and the methods have been updated accordingly (line 837).

10) While the use of flow cytometry to evaluate the cell cycle stage blocked by flavopiridol (G2/M) has been previously published, new here is a correlation to mNG KINF localization and cell morphology. While it can be argued that flavopiridol blocks cell cycle progression at G2/M if mNG marks nascent spindle microtubule formation, or rather G2 alone as the authors argue, there is little new information here. Given the promiscuous inhibitory behavior of flavopiridol, this result is unlikely to be of broad interest.

G2 has never been defined before in *Leishmania* due to the difficulties in distinguishing G2 cells from S and M, and thus our methodology enhances cell cycle analysis of this parasite. While applying this to the understanding of the action of flavopiridol may only have led to an incremental increase in knowledge about its action, our work nevertheless provides proof of principle of the utility of our analysis pipeline to determine the mode of action of cell cycle inhibitors. Further, flavopiridol appears to not have a particularly pleiotropic action in Leishmania as it results in a precise cell cycle block at G2 through inhibition of the mitotic CDK, CRK3 (https://doi.org/10.1016/S0166-6851(01)00220-1) . Knowing where flavopiridol inhibits the Leishmania cell cycle, and the duration of G2 will also permit the design of more precise synchronisation and release experiments to study the short duration events that occur at the end of the cell cycle (G2, M and cytokinesis). Currently, hydroxyurea is more commonly used to synchronise Leishmania in G1/S (with flavopiridol having largely been neglected as a cell cycle synchroniser for this parasite since 2001), but this requires incubations in excess of 6 hrs post-release to study the end of the cell cycle, compared with 2-3 hrs when synchronising with flavopiridol (https://doi.org/10.1371/journal.ppat.1006615). We hope our work will serve as a reminder of an alternative tool for cell cycle studies.

---

6. PLOS authors have the option to publish the peer review history of their article (what does this mean?). If published, this will include your full peer review and any attached files.

If you choose “no”, your identity will remain anonymous but your review may still be made public.

Do you want your identity to be public for this peer review? For information about this choice, including consent withdrawal, please see our Privacy Policy.

Reviewer #1: **Yes:** Danae Schulz

Reviewer #2: No
